# Activator of G-protein signaling 8 is involved in VEGF-induced choroidal neovascularization

**DOI:** 10.1038/s41598-018-38067-4

**Published:** 2019-02-07

**Authors:** Hisaki Hayashi, Abdullah Al Mamun, Masayuki Takeyama, Aya Yamamura, Masahiro Zako, Rina Yagasaki, Tsutomu Nakahara, Motohiro Kamei, Motohiko Sato

**Affiliations:** 1 Department of Physiology, Aichi Medical University, Nagakute, Japan; 2 Department of Ophthalmology, Aichi Medical University, Nagakute, Japan; 3 Department of Ophthalmology, Asai Hospital, Seto, Japan; 4 Department of Molecular Pharmacology, Kitasato University School of Pharmacy, Tokyo, Japan

## Abstract

Choroidal neovascularization (CNV) is associated with age-related macular degeneration (AMD), a major cause of vision loss among elderly people. Vascular endothelial cell growth factor (VEGF) is essential for the development and progression of AMD, and VEGF signaling molecules are effective targets for the treatment of AMD. We recently reported that activator of G-protein signaling 8 (AGS8), a receptor-independent Gβγ regulator, is involved in VEGF-induced angiogenesis in cultured endothelial cells (EC); however, the role of AGS8 in CNV is not yet understood. This study aimed to explore the role of AGS8 in CNV in cultured cells, explanted choroid tissue, and laser-induced CNV in a mouse AMD model. AGS8 knockdown in cultured choroidal EC inhibited VEGF-induced VEGFR-2 phosphorylation, cell proliferation, and migration. AGS8 knockdown also downregulated cell sprouting from mouse choroidal tissue in *ex vivo* culture. A mouse model of laser-induced CNV, created to analyze the roles of AGS8 *in vivo*, demonstrated that AGS8 mRNA was significantly upregulated in choroidal lesions and AGS8 was specifically expressed in the neovasculature. Local AGS8 knockdown in intravitreal tissue significantly inhibited laser-induced AGS8 upregulation and suppressed CNV, suggesting that AGS8 knockdown in the choroid has therapeutic potential for AMD. Together, these results demonstrate that AGS8 plays critical roles in VEGF-induced CNV.

## Introduction

Age-related macular degeneration (AMD) is a major cause of blindness among elderly people in developed countries^1^. It can be categorized into “dry” and “wet” forms^2^. Wet AMD is characterized by the growth of abnormal vessels from the choroid into the subretinal space, known as choroidal neovascularization (CNV). It occurs less frequently than the dry form but is responsible for 90% of cases of acute blindness^3^. Newly formed blood vessels from the choroid usually break through Bruch’s membrane and develop under the retinal pigmented epithelium (RPE)^4^. Development of wet AMD is associated with local production of VEGF, therefore inhibition of VEGF signaling with anti-VEGF agents (i.e., ranibizumab, pegaptanib sodium, and aflibercept) has revolutionized the treatment of AMD via an antiangiogenic potential^5,6^. Although VEGF has essential functions in the pathophysiology of the cardiovascular system, several adverse events have been reported after the administration of anti-VEGF agents, including thromboembolic events, myocardial infarction, stroke, hypertension, gastrointestinal perforations, and kidney disease^7–9^. Therefore, there is an urgent need for alternative approaches to the treatment of CNV.

Heterotrimeric G-protein signaling plays multiple important roles in physiological and pathophysiological conditions including angiogenesis^10^. Conventionally, heterotrimeric G-proteins are activated by G-protein-coupled receptors (GPCR) in response to ligand stimuli; however, it is recognized that they are directly regulated by accessory proteins, which regulate G-protein signaling independently of GPCR activation^11^. We previously identified activator of G-protein signaling (AGS) 8, also known as fibronectin type III domain containing 1, from a repetitive transient ischemia model of the rat heart. AGS8 interacts directly with Gβγ subunits^12^ and plays a pivotal role in hypoxia-induced apoptosis of cardiomyocytes in association with Gβγ^13,14^. Since the rat heart in the transient ischemia model from which we cloned AGS8 has highly developed collaterals^12^, we hypothesized that AGS8 is involved in the regulation of vascular development. Therefore, we further investigated the roles of AGS8 in vascular formation and found that knockdown of AGS8 by small interfering RNA (siRNA) reduced cell surface localization of AGS8 and inhibited VEGF-induced tube formation, cell growth and migration, as well as the phosphorylation status of signaling molecules including VEGFR-2, ERK1/2, and p38/MAPK. Furthermore, AGS8-Gβγ formed a protein complex with VEGFR-2, suggesting a potential role for AGS8-Gβγ in VEGF signal processing during angiogenesis^15^. In addition, we recently demonstrated that AGS8 is also involved in the regulation of VEGFC-induced lymphangiogenesis through VEGFR-3 regulation in lymphatic endothelial cells^16^ but is not involved in FGF- or EGF-induced cellular events^15,16^, suggesting that AGS8 is a specific VEGF signaling regulator.

To date, however, the pathophysiological significance of AGS8 remains unclear in *in vivo* animal models. Here, to elucidate the potential involvement of AGS8 in CNV, the roles of AGS8 were examined with an *in vitro* choroidal endothelial cell culture, *ex vivo* choroid explant culture, and an *in vivo* murine CNV model, which is an established model that closely mimics the pathogenesis of human AMD. We demonstrate here for the first time that AGS8 is involved in the development of CNV and is a potential therapeutic target for AMD.

## Results

### Inhibition of AGS8 attenuates VEGF-induced cellular events in RF/6A choroidal endothelial cells

**Figure 1:**
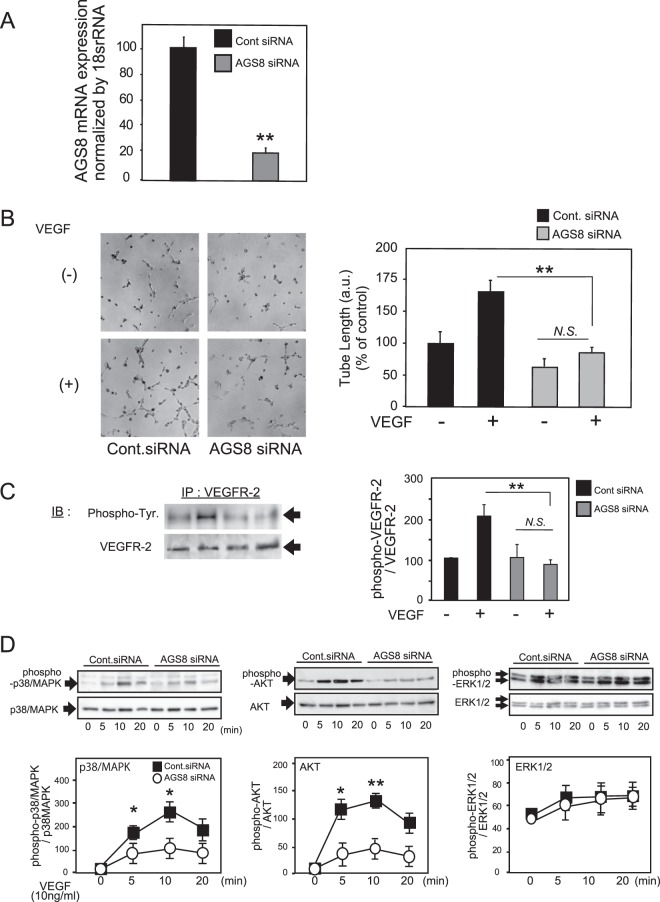
AGS8 knockdown inhibited VEGF-induced tube formation and the phosphorylation of VEGFR-2 in choroid endothelial cells. (**A**) Evaluation of AGS8 knockdown by real-time PCR. RF/6A cells were transfected with control siRNA or AGS8 siRNA#1. After 48 h, AGS8 mRNA expression was analyzed by real-time PCR assay. Data are expressed as means ± standard error of the mean (s.e.m) from four experiments performed in triplicate. ^**^P < 0.01 (unpaired *t-*test). (**B**, left panel) Tube formation analysis of AGS8 knockdown in RF/6A cells. Cells were transfected with control siRNA or AGS8 siRNA#1, and seeded on Matrigel coated plates. Cells were treated with VEGF at 10 ng/mL and maintained for 6 h, and images were taken. (**B**, right panel) Tube length was digitally quantified. Data are means ± s.e.m. from four independent triplicate experiments. ^**^P < 0.01 (two-way ANOVA with Tukey’s correction). N.S., not significant (P ≥ 0.05; unpaired t-test). (**C**) Phosphorylation of VEGFR-2 in RF/6A cells. RF/6A cells were transfected with control siRNA or AGS8 siRNA#1, then cultured for 48 h, starved and stimulated with 25 ng/mL VEGF for 10 min. Cell lysates were immunoprecipitated with VEGFR-2 antibody and subjected to immunoblotting with phosphotyrosine or VEGFR-2 antibodies. Data are means ± s.e.m. from four independent experiments. ^**^P < 0.01 (two-way ANOVA with Tukey’s correction). N.S., not significant (unpaired t-test). Full-size images of immunoblots are presented in Supplementary Fig. 1. (**D**) Effect of AGS8 knockdown on the VEGF-induced phosphorylation of p38/MAPK, AKT, and ERK(1/2) in RF/6A cells. At 48 h after transfection of control siRNA or AGS8 siRNA#1, the cells were stimulated with VEGF at 10 ng/mL, and cell lysates (10–20 μg) were subjected to immunoblotting. Developed digital images were quantified by densitometric analysis. The values were normalized by p38/MAPK, AKT, and ERK(1/2). ^*^P < 0.05, ^**^P < 0.01 (two-way ANOVA with Tukey’s correction). Data are the mean ± s.e.m from 4 independent experiments. Full-size images of immunoblots are presented in Supplementary Fig. 2.

To examine the role of AGS8 in CNV, we first examined the effect of AGS8 knockdown in cultured choroidal endothelial cells, RF/6A cells, which originate from rhesus choroid/retina tissues and are frequently used for *in vitro* CNV analyses^17–19^. Transfection of RF/6A cells with siRNA successfully inhibited the expression of AGS8 mRNA (18.5 ± 3.2% versus control (mean ± s.e.m); ^**^P < 0.01; Fig. 1A). Since VEGF is a primary factor necessary for the initiation of sprouting angiogenesis by binding to VEGFR-2^20^ and is known to play a crucial role in pathological angiogenesis, CNV and wet AMD, the effects of AGS8 knockdown in VEGF-induced cellular events in choroidal ECs were analyzed. VEGF-induced tube formation of RF/6A cells on Matrigel was significantly attenuated by AGS8 siRNA#1 (55.2 ± 2.9%; ^**^P < 0.01; Fig. 1B).

AGS8 knockdown also inhibited VEGF-induced phosphorylation of VEGFR-2 (44.1 ± 2.3%; ^**^P < 0.01; Fig. 1C), determined by immunoprecipitation with a specific VEGFR-2 antibody followed by immunoblotting with anti-phosphotyrosine antibody. The effect of AGS8 knockdown on the downstream signaling pathway of VEGFR-2 was examined. VEGF induced the phosphorylation of p38MAPK and AKT with a peak at 10 min (Fig. 1D). Knockdown of AGS8 inhibited the phosphorylation of p38/MAPK and AKT. (Fig. 1D).

**Figure 2:**
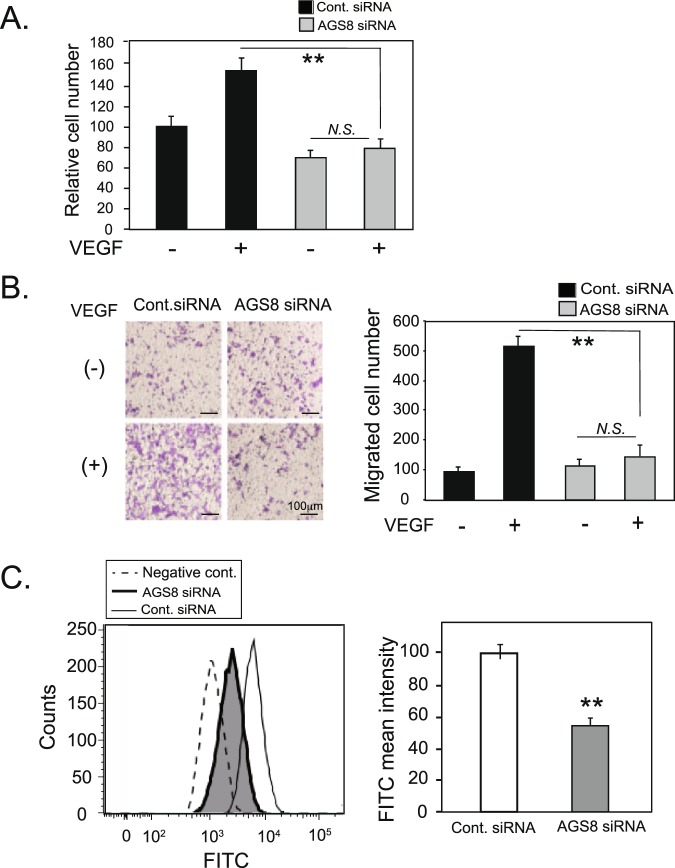
AGS8 knockdown inhibited VEGF-induced cell proliferation and cell migration in choroid endothelial cells. (**A**) MTT assay of AGS8 knockdown in VEGF-stimulated cells. After transfection of control siRNA or AGS8 siRNA#1, cells were stimulated by VEGF at 10 ng/mL for 48 h and cell proliferation was analyzed with MTT assay. Data are means ± s.e.m. from 6 independent experiments repeated 4 times. ^**^P < 0.01 (two-way ANOVA with Tukey’s correction). N.S., not significant (unpaired t-test). (**B**) Transwell migration analysis of AGS8 knockdown in VEGF-stimulated cells. Cells transfected with AGS8 siRNA#1 were seeded in transwells, stimulated by VEGF at 10 ng/mL for 4 h, and stained. Images of migrated cells were taken in three random microscopic fields. Data are means ± s.e.m from four independent triplicate experiments. ^**^P < 0.01 (two-way ANOVA with Tukey’s correction). N.S., not significant (unpaired t-test). (**C**) VEGFR-2 expression on the cell surface is reduced by AGS8 knockdown. RF/6A cells were transfected with control siRNA or AGS8 siRNA#1, and labeled with an fluorescein-conjugated anti-VEGFR-2 antibody, and flow cytometric analyses were performed as described in the Methods. The histograms represent cell counts (Y-axis, linear scale) versus fluorescein intensity (X-axis, log scale). Negative control indicates negative control cells treated with respective isotype human IgG labeled with fluorescein. Fluorescence mean intensity obtained from the histograms was quantified and shown in bar graphs (right panel). Data are the mean ± s.e.m from 4 experiments. ^**^P < 0.01 versus control siRNA-treated cells (unpaired t-test).

The influence of AGS8 knockdown on VEGF-mediated cellular events was further analyzed. VEGF-induced proliferation of cells was inhibited by AGS8 knockdown (52.6 ± 3.1% versus control; ^**^P < 0.01; Fig. 2A) determined by MTT assay. VEGF-induced migration of cells was also attenuated by AGS8 knockdown (21.6 ± 5.1% versus control; ^**^P < 0.01; Fig. 2B) determined by a transwell migration assay. Two other siRNAs targeting different segments of AGS8 reduced the expression of AGS8 mRNA and inhibited the VEGF-stimulated growth of RF/6A cells (Fig. S3A,B). These data suggest that AGS8 is involved in VEGF-mediated cellular events in choroidal ECs.

The effect of AGS8 knockdown on VEGFR-2 signal is associated with a decrease of cell surface expression of VEGFRs^15,16^. Therefore, we analyzed the cell surface expression of VEGFR-2 in RF/6A cells using FACS analysis with an FITC-conjugated anti-VEGFR-2 antibody. VEGFR-2 at the cell surface was significantly decreased by AGS8 siRNA#1 (43.3 ± 3.9% vs. control siRNA, mean ± s.e.m., ^*^P < 0.01; Fig. 2C). Two other AGS8 siRNAs also reduced the cell surface expression of VEGFR-2 in RF/6A cells (Fig. S3C).

### Inhibition of AGS8 attenuates cell sprouting in mouse choroid explant culture

**Figure 3:**
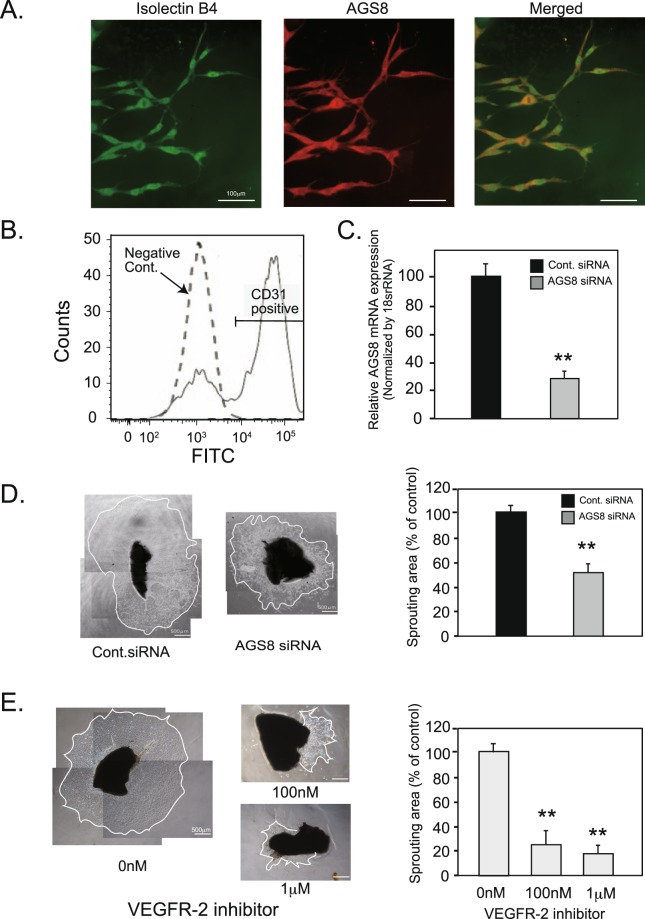
The effect of AGS8 knockdown in cells sprouting from explant cultures of murine RPE-choroid. (**A**) AGS8 expression was determined by immunofluorescence staining of sprouting cells from explants. Sprouting cells on a coverslip were fixed with 4% PFA and stained with an anti-AGS8 antibody (red) and fluorescein labeled isolectin B4 (Vector Laboratories, Burlingame, CA))(green). Images were obtained with a fluorescence microscope. Representative images are shown from 10 specimens in 2 independent experiments with similar results. Scale bars are 100 μm. (**B**) The ratio of CD31-positive cells sprouting from choroid explant culture was assessed. Mouse choroid was isolated and cut into pieces and cultured on Matrigel-coated cover glass. Sprouting cells were collected on day 5 and stained with CD31-FITC antibody before processing for flow cytometric analysis. Negative control indicates cells stained with control IgG-FITC. (**C**) For AGS8 knockdown, AGS8 siRNA#1 was transfected to explanted cultures on days 2 and 3, and AGS8 mRNA expression on day 4 was determined by real-time PCR. Data are means ± s.e.m from four time-independent quaternary experiments. ^**^P < 0.01 (unpaired t-test). (**D**, left panel.) Images were taken on day 4 after AGS8 knockdown in sprouting cells from choroidal explanted cultures. (**D**, right panel) The areas of cells sprouting on day 4 were quantified by ImageJ software. Data are means ± s.e.m. from five time-independent experiments. ^**^P < 0.01 (unpaired t-test). (**E**) The explants were treated with vehicle or VEGFR-2 inhibitor for 4 days. Images were taken and the area of cell sprouting was quantified by ImageJ software. Data are the mean ± s.e.m. from 12 explants in 2 time-independent experiments with similar results. ^**^P < 0.01 (unpaired t-test).

Next, we examined the role of AGS8 in a choroid sprouting assay, which is an *ex vivo* experimental model of CNV. Sprouting of vascular ECs from the choroid explant reproduces the processes of microvascular angiogenesis, including cell proliferation, cell migration, and tube formation^21^. Mouse choroid was dissected from the retina, and the fragments were embedded in Matrigel and cultured for 4 days. The cells growing out of the explants were stained with the endothelial marker isolectin and AGS8 (Fig. 3A). Flow cytometric analysis indicated that almost 70% of cells spreading out from the explant were CD31-positive endothelial cells (70.1% ± 2.04, mean ± s.e.m, n = 4) (Fig. 3B), which was consistent with a previous report^21^. To analyze its role, AGS8 was knocked down by siRNA transfection of the explants at days 2 and 3 of culture, and the culture was continued up until day 4. Real-time polymerase chain reaction (PCR) showed that transfection of AGS8 siRNA attenuated the expression of AGS8 in the migrated cells (24.2 ± 4.1% versus control; Fig. 3C). Finally, the area occupied by migrated cells was digitally quantified; it was found that an area of cells sprouting out from the explant was significantly reduced by AGS8 knockdown (54.2 ± 5.7% versus control, ^**^P < 0.01; Fig. 3D). The reduction of the sprouting area by AGS8 siRNA probably reflects AGS8 knockdown in endothelial cells; however it may include additional effects of AGS8 knockdown in other cells such as pericytes and fibroblasts. Separate experiments indicated that a VEGFR-2 inhibitor significantly attenuated the outgrowth of cells from explants in a dose-dependent manner (Fig. 3E), suggesting that AGS8 is involved in the regulation of VEGFR-2 signaling, which is critical for the initiation of microvascular angiogenesis in the choroid.

### Induction of AGS8 expression in a laser-induced mouse CNV model

**Figure 4:**
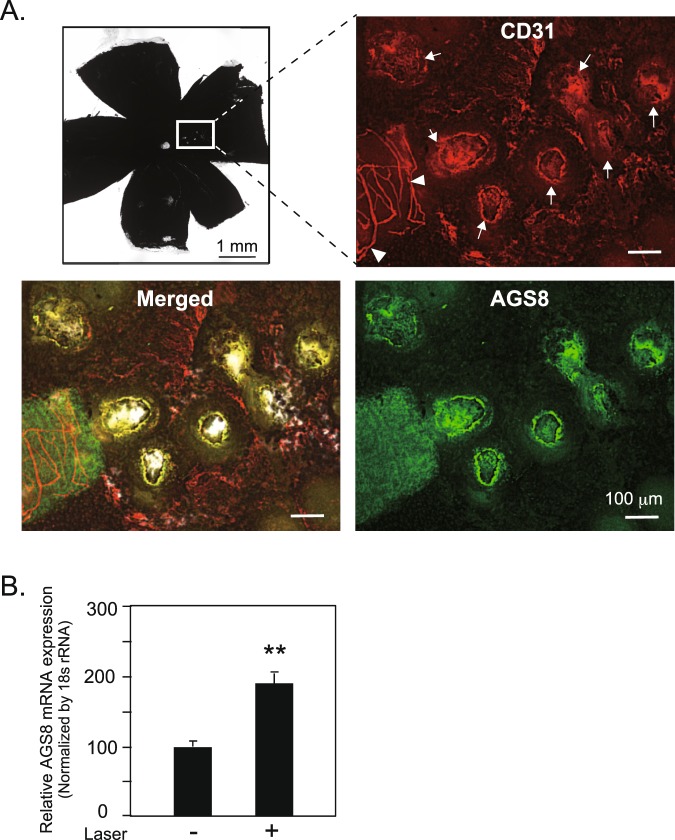
Upregulation of AGS8 mRNA expression in choroidal EC in a laser-induced CNV experiment. (**A**) Picture of the flat-mounted choroid 2 days post-laser-induced CNV. Mouse retinas in each eye were subjected to laser photocoagulation for induction of experimental CNV with an argon laser photocoagulator. Dissected mouse RPE-choroid was immunostained with CD31 (red; Cy3) and AGS8 (green; Alexa488) antibodies. The arrow indicates CD31-positive cells in the laser-induced CNV area, whereas the arrowhead indicates CD31-positive cells outside of the laser-induced lesions. (**B**) AGS8 mRNA expression in CNV area. Two days after laser-induced CNV, mouse eyes were dissected, RPE-choroid was isolated, the lesion area was dissected, and total RNA was purified. Real-time PCR was performed to evaluate laser-induced AGS8 mRNA expression. Data are means ± s.e.m from four time-independent experiments. ^*^P < 0.05 (unpaired t-test).

The laser-induced CNV model is widely used as model of AMD with pathological angiogenegsis^22,23^. Two days after argon laser-induced injury of Bruch’s membrane, mouse eyes were dissected and the choroidal tissues associating with RPE were isolated. CNV induction was evaluated by immunostaining with CD31 (endothelial marker; red in Fig. 4A) and AGS8 (green) antibodies. CD31 positive cells were observed in the laser-induced lesion, indicating newly formed vessels originating from the preexisting choroidal vessels (Fig. 4A, arrow). Interestingly, AGS8 was identified in CD31-positive cells in the laser-induced CNV (Fig. 4A, arrows), but not detectable in other areas nor CD-31 positive pre-existing vasculature (Fig. 4A, arrowheads), suggesting the specific localization of AGS8 in angiogenic endothelial cells. RPE-choroid tissue surrounding the laser-induced lesion, or intact RPE-choroid in a corresponding area, were dissected and subjected to real-time PCR on day 2 to detect the expression of AGS8 mRNA. The relative expression of AGS8 mRNA was significantly higher in CNV lesions than in the control (191.2 ± 9.1% versus control, P < 0.01; Fig. 4B). These results show that AGS8 was expressed in newly formed vessels in a laser-induced AMD model mouse.

### Suppression of CNV in AGS8 knockdown in a mouse CNV model

**Figure 5:**
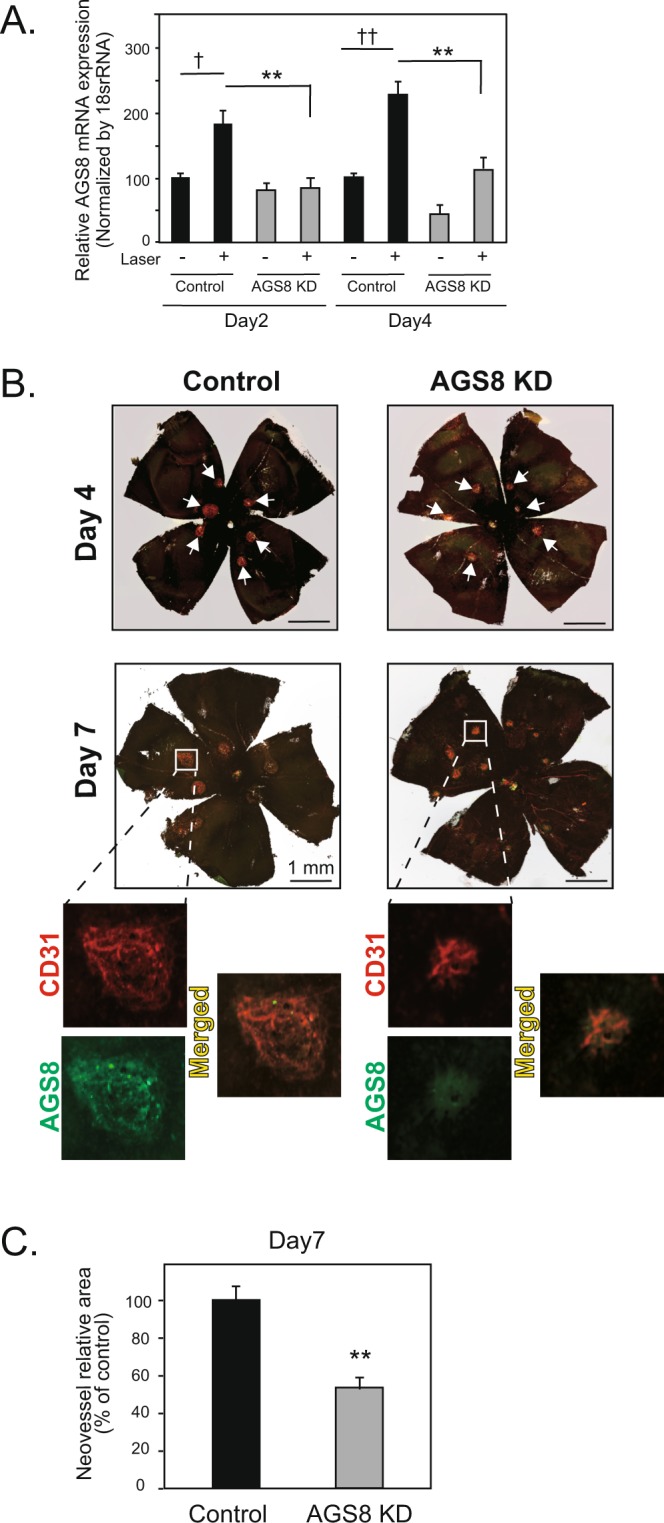
Suppression of laser-induced CNV in AGS8 knockdown. (**A**) Expression of AGS8 mRNA in CNV tissue. At 2 or 4 days after laser-induced CNV surgery and intravitreal siRNA injection, mouse eyes were dissected, choroid was isolated, total RNA was purified, and real-time PCR was performed to evaluate the effect of AGS8 siRNA#1-induced knockdown (AGS8KD). Data are means ± s.e.m from four time-independent triplicate experiments. ^†^P < 0.05, ^††^P < 0.01 (unpaired t-test), **P < 0.01 (two-way ANOVA with Tukey’s correction). (**B**) SiRNA was injected into the vitreous for AGS8 knockdown in the choroid following laser-induced CNV surgery. Newly formed vessels were visualized on days 4 and 7 by immunohistochemical staining with CD31 (red) and AGS8 (green) antibodies. Representable images were shown. (**C**) Quantification of vascularized area obtained from (**B**). Data are shown as means ± s.e.m. (N = 8 in each group, ^**^P < 0.01, unpaired t-test).

Next, we attempted to knockdown AGS8 in the choroidal tissue by direct injection of siRNA into the vitreous^24^. After laser induction into mouse eyes,  20 ng of AGS8 siRNA or control siRNA combined with transfection reagent were concentrated to 1 µL and delivered to the intravitreal body by injection. AGS8 mRNA expression was significantly upregulated in CNV lesions (laser+) compared with control tissue (laser−) with control siRNA on day 2 (181 ± 18.0%) and day 4 (221 ± 18.5%; †† < 0.01, † < 0.05; Fig. 5A) and significantly attenuated by AGS8 siRNA on day 2 (43.9 ± 7.2% versus control) and day 4 (48.2 ± 10.7% versus control; **P < 0.01). Either 4 or 7 days after laser-induction, RPE-choroid tissues were isolated and co-immunostained with CD31 and AGS8 antibodies, indicating that the AGS8 expression observed in CNV was reduced by AGS8 knockdown (Fig. 5B). Interestingly, the CD31-postive area in the CNV, which was co-localized with AGS8, was attenuated by AGS8 knockdown (Fig. 5B). Neovessel areas measured on day 7 indicated that AGS8 knockdown significantly prevented the formation of CNV (56.2 ± 3.9% versus control; ^**^P < 0.01; Fig. 5C). Together, these results demonstrate that AGS8 was involved in the formation of CNV in a mouse AMD model and AGS8 loss of function potentially inhibits AMD by preventing or ameliorating CNV.

## Discussion

In this study, we demonstrated novel regulation of CNV via an accessory protein for G-proteins, AGS8. Knockdown of AGS8 in the cultured choroidal cells inhibited VEGF-induced tube formation on Matrigel and the phosphorylation of VEGFR-2, as well as VEGF-induced cell migration and cell proliferation. In an *ex vivo* mouse choroid explant culture model, AGS8 knockdown significantly inhibited endothelial cell sprouting. In the laser-induced mouse AMD model, AGS8 was induced in neovessels on days 2 and 4 after surgery. Interestingly, intravitreal AGS8 siRNA injections significantly inhibited CNV formation and the vascular budding area of the RPE-choroid complex. These findings complemented the *in vitro* study, which showed that the molecular mechanism of angiogenesis is mediated by AGS8^15^ and demonstrated the regulation of angiogenesis *in vivo* by accessory proteins for G-protein. Our data also suggest the potential of AGS8 as a therapeutic target to control neovascularization in human diseases.

The mechanisms of CNV on AMD are complicated and have not yet been clarified^25^. It is now well known that VEGF plays a crucial role in abnormal blood vessel development in CNV^26^ and that the inhibition of VEGF signaling can effectively control angiogenesis. In fact, intravitreal injections of anti-VEGF agents pegaptanib and ranibizumab have currently been approved for AMD treatment, while off-label use of bevacizumab has also become common^26^. Since VEGFR-2 is essential in almost all VEGF-mediated responses in pathological angiogenesis^27–29^, apatinib, a VEGFR-2 inhibitor, also effectively inhibits CNV—at least in mice^30^. We previously demonstrated that AGS8 regulated VEGF signaling via VEGFR-2 regulation in vascular endothelial cells *in vitro*^15^. Therefore, we attempted to control angiogenic events in an *in vivo* animal model through the suppression of AGS8. AGS8 knockdown successfully exerted anti-VEGF effects by preventing VEGF-mediated signaling, which led to the suppression of CNV in a mouse AMD model. This observation provides additional information on how to control the development of CNV.

Anti-VEGF therapies targeting VEGF have become integral components of anticancer regimens for many tumor types^31^ and ocular diseases such as diabetic retinopathy^32^ and AMD. Intravitreal injection of anti-VEGF agents has revolutionized the treatment of AMD, and these agents have been reported as highly effective for improving visual function. However, because VEGF is involved in a wide variety of physiological process, anti-VEGF agents carry potential risks of adverse events. Repeated and long-term injections of anti-VEGF agents may increase the chance of the systemic complications of thromboembolic events, myocardial infarction, stroke, hypertension, gastrointestinal perforations, and kidney disease^7–9^. Thus, AGS8 targeting could be an alternative approach for the specific downregulation of VEGF signaling in CNV along with fewer adverse effects because its expression was induced after laser-induced CNV and was limited in the neovasculature in the laser-induced lesion, not in the pre-existing vasculature.

AGS8 is an accessory protein for heterotrimeric G-proteins isolated from a repetitive transient ischemia model of the rat heart with extensive collateral development^12^. AGS8 interacts with Gβγ subunits and regulates cellular function without the activation of GPCRs^12,13^. Our previous study demonstrated that AGS8 is involved in the trafficking of VEGFR-2 to the cell surface in endothelial cells. Furthermore, the expression of c-terminal AGS8 increases the expression of VEGFR-2 in cultured cells^15^, which might due to the stabilization of VEGFR-2 in the AGS8-Gβγ protein complex. Recently, it was reported that VEGF mRNA and protein expression are induced in RPE cells and the receptor VEGFR-2 is also present in choroidal ECs in a laser-induced murine AMD model^33^. Here, AGS8 was induced into the choroidal ECs in the neovasculature and might have contributed to development of CNV in choroidal ECs by regulating the VEGF signaling pathway.

In summary, the information we provide here is novel. AGS8 expression was upregulated in the CNV no later than 2 days after laser induction, and the inhibitory effect of AGS8 knockdown was significant when the CNV began to form. Restriction of AGS8 expression to within the neovasculature suggested that targeting AGS8 may be advantageous because it would not cause off-target effects in pre-existing vessels. This new information contributes to our understanding of CNV in human AMD.

## Methods

### Materials

Double-stranded siRNA oligonucleotides to rhesus and mouse siRNA (AGS8siRNA#1: sense, 5′-CCCUGCCUGCCAGCUUUCAU-3′; AGS8siRNA#2: sense, 5′-GAAGCCAAGGCACGTGAAACT-3′; AGS8siRNA#3: sense, 5′-GTGAAACTGCTGTCCACTAAA-3′), negative control with scrambled sequences, and Lipofectamine siRNA Max reagent were purchased from Life Technologies (Carlsbad, CA). Antibodies against VEGFR-2 (#2479; 1:1000 dilution for immunoblot, 1:100 dilution for immunoprecipitation), Anti-AKT (#9272), anti-phospho-AKT (Ser473) (#9271), anti-p44/42 MAPK (#9102), anti-phospho-p44/42 MAPK (Thr202/204) (#9101), anti-p38MAPK (#9212), anti-phosphop38MAPK (Thr180/Tyr182) (#9211) were purchased from Cell Signaling Technology (Danvers, MA), and anti phosphotyrosine (PY20; 1:2000 dilution for immunoblot) was obtained from Santa Cruz Biotechnology (Dallas, TX). RF6A cells were obtained from Riken Bioresource Center (Tsukuba, Japan). RF/6A is a immortal endothelial cell line derived from the choroid-retina of a rhesus macaque^34^, which has been used in biological studies of endothelial cells^17–19^. VEGFR-2 inhibitor was purchased from R&D Systems (Minneapolis, MN).

### Animals

C57BL/6N mice were purchased from Japan SLC, Inc. (Hamamatsu, Japan). All animal procedures were approved by The Animal Care and Use Committee of Aichi Medical University (2017-88) and performed in accordance with the Research Guideline for Animal Experimentation of Aichi Medical University. Mice were housed in a room maintained at 23 ± 1 °C with a 12 h light-12 h dark cycle and had free access to food and water.

### Choroidal cell culture and siRNA transfection

The choroid-retinal endothelial cell line RF/6A cells were cultured in Dulbecco’s Modified Eagle’s Medium (DMEM; Wako Chemicals, Osaka, Japan), supplemented with 10% fetal bovine saline (FBS) (Sigma-Aldrich, St. Louis, MO) and penicillin-streptomycin (Wako Chemicals). For siRNA transfection, RF/6A cells were cultured in six-well plates to subconfluence and treated with 40 nM rhesus AGS8 siRNA or control siRNA with Lipofectamine siRNA Max (Thermo Fisher Scientific., Waltham, MA) for 48 h according to the manufacturer’s instructions.

### Tube formation assay

Briefly, 96-well plates were coated with 50 μL of growth-factor-reduced Matrigel (BD Biosciences, San Jose, CA), and polymerized at 37 °C for 1 h. RF/6A cells were starved using DMEM with 1% FBS for 6 h, trypsinized and then suspended in 250 μL of DMEM with 1% FBS. Cells were seeded at 1 × 10^4^ cells/well onto Matrigel and again incubated. Under some conditions, cells were stimulated with 10 ng/mL of VEGF (R&D Systems, Minneapolis, MN). Images were taken at 6 h, and the tube length was determined by the use of ImageJ software^35^. The total tube length of each image was determined by an observer blinded to the conditions. Data are presented in arbitrary units.

### MTT assay

Cell proliferation was analyzed using a Vybrant MTT cell proliferation assay kit (Life Technologies), according to the product manual. Briefly, cells in 6-well plates were transfected with siRNAs and incubated for 24 h. Cells were transferred to 96-well plates at 5 × 10^3^ cells/well. After 24 h, cells were starved with DMEM containing 1% FBS for 6 h and treated with VEGF for 48 h. Cells were then incubated with MTT at 37 °C for 4 h. Medium was removed, and SDS-HCl solution (10% SDS, 0.01 M HCl) was added to each well. Absorbance was measured with a microplate reader after incubation for 18 h at 37 °C.

### Cell migration assay

RF/6A cells suspended at 1 × 10^4^ cells/200 μL were seeded on fibronectin coated Transwell inserts with 8 μm pores (BD Biosciences) pre-coated with 3% bovine serum albumin, then stimulated with 10 ng/mL of VEGF in the lower chamber. After 4 h, the Transwell inserts were fixed in 4% PFA and stained with 1% crystal violet. Digital microscopic images of the underside of each Transwell insert were taken at three independent microscopic fields per transwell.

### Flow cytometric analysis

Fluorescence-activated cell sorting (FACS) was performed using a FACS Canto II (BD Biosciences, San Jose, CA) as described previously^15^. RF/6A cells were washed with PBS, harvested with Accutase (Innovative Cell Technologies, San Diego, CA), and fixed with 2% PFA for 30 min. The cells were washed with PBS containing 1% FBS, and labeled with a fluorescein-conjugated anti-human VEGFR-2 antibody (1:100 dilution) or fluorescein-conjugated IgG1 isotype control (1:100 dilution; IC002F; R&D Systems, Minneapolis, MN) for 1 h in the dark on ice. The data were analyzed by FACS DIVA (BD Biosciences) or FlowJo software (FlowJo LLC, Ashland, OR).

### Laser-induced experimental CNV Model

Eight-week-old C57BL6/N male mice were used for laser-induced CNV induction according to a previous report^36^. Briefly, after anesthesia was induced with intraperitoneal injection of 1% pentobarbital sodium (50 mg/kg; Kyoritsu Seiyaku, Tokyo, Japan), the pupils were dilated using 0.5% tropicamide and 0.5% phenylephrine (Santen Pharmaceuticals, Osaka, Japan), followed by topical anesthesia using 0.4% oxybuprocaine hydrochloride (Santen Pharmaceuticals). Bruch’s membrane was injured under a slit-lamp microscope with an argon laser photo coagulator using the following parameters: 514 nm wavelength, 200 mW intensity, 0.1 s duration, and a 100 μm lesion diameter. Equal numbers of laser lesions were generated in the control eye and AGS8 knockdown eye (6–8 spots per eye) in every preparation. On specific days indicated, choroids were isolated and processed for the following experiments after laser induction.

### Intravitreal injection of siRNA to knockdown gene expression in choroid

Experiments were essentially performed as previously reported^24^. Briefly, 100 ng of siRNA was combined with 4.5 μL of Transit-TKO (Takara Bio, Kyoto, Japan) in 50 μL of H_2_O and incubated for 20 min at room temperature. The mixed solution was evaporated under vacuum and the precipitate was dissolved in 5 μL H_2_O. Then, 1 μL of concentrated reagent mixture was injected into the vitreous using a Hamilton syringe with a 32 gauge needle.

### Explant culture of choroid and siRNA transfection

Sprouting of choroidal layers was tested as previously described^21^. Briefly, 8-week-old C57BL/6n male mouse eyes were enucleated immediately after euthanasia. Choroid tissue was isolated and cut into pieces of approximately 1 mm × 1 mm fragments and placed on a coverslip coated with growth factor-reduced Matrigel (20 μL/well; BD Biosciences) in 24-well plates. The plates were then incubated at 37 °C for about 10 min to allow the Matrigel to solidify. Five hundred microliters of EGM-2 (Lonza, Basel, Switzerland) containing VEGF (0.5 ng/mL) was added to each well of the plate and incubated in 5% CO_2_. Images were taken using a phase contrast microscope and merged for further analysis. For siRNA transfection, the explants were transfected with 40 nM mouse AGS8 siRNA or control siRNA with Lipofectamine siRNA Max at days 2 and 3 and cultured until day 4.

### Real-time PCR

Isolation of total RNA from cultured RF/6A cells and explant cultures of choroidal tissue and rat choroidal lesions were performed with an RNeasy Mini Kit (Qiagen, Valencia, CA) and cDNA synthesis with ReverTra Ace (Toyobo, Osaka, Japan), respectively. Real-time PCR was carried out with KAPA SYBR Fast qPCR kit (Nippon Genetics, Tokyo, Japan) and STEP one plus (Bio-Rad) according to the manufacturers’ instructions. The primers for real-time PCR were as follows: mouse AGS8 forward, 5′-TCTGGACACACGACTTCTGC-3′; reverse, 5′-GCTGTCTCTCCCATTTGGCT-3′; primer sequences for rhesus AGS8, which agrees with human primer sequence designed previously^15^, were forward, 5′-TTCCGTAACCCTCTCTCCCG-3′; and reverse, 5′-AACCCACGATCAAGGTCCAC-3′.

### Immunohistochemistry

The explants were cultured on glass coverslips (Matsunami Glass, Osaka, Japan) coated by matrigel. The explants with sprouting cells cultured for 4 days were fixed with 4% PFA for 5 min, and choroid explants were removed. Following washing with PBS, the cells sprouted were incubated with 5% normal donkey serum in PBS for 1 h. The cells were incubated with AGS8 antibody13 and fluorescein labeled isolectin B4 (Vector Laboratories, Burlingame, CA) for 18 h, washed with PBS, incubated by HRP-labeled anti-rabbit IgG antibody for 1 h, and followed by TSA signal amplification system (Perkin Elmer, Shelton, CT) for AGS8 detection. The coverslips were mounted on a slide glass (Matsunami Glass) with VECTASHIELD mounting medium (Vector Laboratories, Burlingame, CA, USA). Images were obtained under an LSM710 fluorescence microscope (Zeiss, Oberkochen, Germany).

Immunohistochemistry of RPE-choroidal flat-mounts was performed as described previously^36^. Mouse eyes were enucleated and fixed in 4% PFA for 30 min on ice. After the cornea, lens, retina and sclera were removed from RPE-choroid in ice-cold PBS, 100% methanol was substituted for PBS for fixation and the tissues were preserved at −20 °C in a freezer. On the day of experiments, methanol was replaced by PBS and 0.5% Triton X (PBST) at room temperature, and PBST with 5% goat serum (Jackson ImmunoResearch, West Grove, PA) blocking buffer for 30 min, before incubation with anti-mouse CD31 antibody (rat, 1:500; Thermo Fisher), or anti-mouse/human AGS8 antibody^13^ (rabbit, 1:200) overnight at 4 °C. The next day, secondary antibodies, Cy3-conjugated rat anti-rat (1:500; Jackson ImmunoResearch) or Alexa-488 conjugated goat anti-rabbit (1:500; Invitrogen) were incubated for 3 h at room temperature. RPE-choroid was washed with PBST, cut by a razor blade to make flat mounted tissue, and the tissues were covered with a thin layer of VectaMount (Vector Laboratories, Burlingame, CA) and a coverslip before examination with a fluorescence microscope, Keyence BZ-9000 (Osaka, Japan).

### Statistical analysis and miscellaneous procedures

All experiments were performed in duplicate, triplicate, or greater and repeated independently at least four times. All statistical analyses were performed with IBM SPSS software. Data were analyzed with one-way or two-way ANOVA followed by post-hoc tests for comparisons of the means. In the case of comparisons in pairs, unpaired t-tests were used. Statistically significant differences between the control and test groups were evaluated if the P value was lower than 0.05 (*) or 0.01 (**) as indicated on the graphs. Error bars in the graphs denote standard errors of the mean (s.e.m). Other miscellaneous procedures have been previously described^37^.

## Supplementary information

Supplementary information

## References

[1] Wong WL (2014). Global prevalence of age-related macular degeneration and disease burden projection for 2020 and 2040: a systematic review and meta-analysis. The Lancet. Global health.

[2] Ambati J, Fowler BJ (2012). Mechanisms of age-related macular degeneration. Neuron.

[3] Hernandez-Zimbron LF (2018). Age-Related Macular Degeneration: New Paradigms for Treatment and Management of AMD. Oxidative medicine and cellular longevity.

[4] van Lookeren Campagne M, LeCouter J, Yaspan BL, Ye W (2014). Mechanisms of age-related macular degeneration and therapeutic opportunities. The Journal of pathology.

[5] Yonekawa Y, Kim IK (2014). Clinical characteristics and current treatment of age-related macular degeneration. Cold Spring Harbor perspectives in medicine.

[6] Balaratnasingam C, Dhrami-Gavazi E, McCann JT, Ghadiali Q, Freund KB (2015). Aflibercept: a review of its use in the treatment of choroidal neovascularization due to age-related macular degeneration. Clinical ophthalmology (Auckland, N.Z.).

[7] Gordon MS, Cunningham D (2005). Managing patients treated with bevacizumab combination therapy. Oncology.

[8] Thulliez M, Angoulvant D, Pisella PJ, Bejan-Angoulvant T (2018). Overview of Systematic Reviews and Meta-analyses on Systemic Adverse Events Associated With Intravitreal Anti-Vascular Endothelial Growth Factor Medication Use. JAMA ophthalmology.

[9] Tunon J (2009). Cardiovascular risk and antiangiogenic therapy for age-related macular degeneration. Survey of ophthalmology.

[10] O’Hayre M, Degese MS, Gutkind JS (2014). Novel insights into G protein and G protein-coupled receptor signaling in cancer. Current opinion in cell biology.

[11] Sato M (2013). Roles of accessory proteins for heterotrimeric G-protein in the development of cardiovascular diseases. Circulation journal: official journal of the Japanese Circulation Society.

[12] Sato M (2006). Identification of a receptor-independent activator of G protein signaling (AGS8) in ischemic heart and its interaction with Gbetagamma. Proceedings of the National Academy of Sciences of the United States of America.

[13] Sato M (2009). Activator of G protein signaling 8 (AGS8) is required for hypoxia-induced apoptosis of cardiomyocytes: role of G betagamma and connexin 43 (CX43). The Journal of biological chemistry.

[14] Sato M (2014). Protection of cardiomyocytes from the hypoxia-mediated injury by a peptide targeting the activator of G-protein signaling 8. PloS one.

[15] Hayashi H, Al Mamun A, Sakima M, Sato M (2016). Activator of G-protein signaling 8 is involved in VEGF-mediated signal processing during angiogenesis. Journal of cell science.

[16] Sakima M, Hayashi H, Mamun AA, Sato M (2018). VEGFR-3 signaling is regulated by a G-protein activator, activator of G-protein signaling 8, in lymphatic endothelial cells. Experimental cell research.

[17] Dong X (2011). Influence of Dll4 via HIF-1alpha-VEGF signaling on the angiogenesis of choroidal neovascularization under hypoxic conditions. PloS one.

[18] Chen S (2018). Anti-neovascularization effects of DMBT in age-related macular degeneration by inhibition of VEGF secretion through ROS-dependent signaling pathway. Molecular and cellular biochemistry.

[19] Feng Y (2018). miR-539-5p inhibits experimental choroidal neovascularization by targeting CXCR7. FASEB journal: official publication of the Federation of American Societies for Experimental Biology.

[20] Chappell JC, Wiley DM, Bautch VL (2011). Regulation of blood vessel sprouting. Seminars in cell & developmental biology.

[21] Shao Z (2013). Choroid sprouting assay: an *ex vivo* model of microvascular angiogenesis. PLoS One.

[22] Schnabolk G, Beon MK, Tomlinson S, Rohrer B (2017). New Insights on Complement Inhibitor CD59 in Mouse Laser-Induced Choroidal Neovascularization: Mislocalization After Injury and Targeted Delivery for Protein Replacement. Journal of ocular pharmacology and therapeutics: the official journal of the Association for Ocular Pharmacology and Therapeutics.

[23] Wang, L. *et al*. miR-126 Regulation of Angiogenesis in Age-Related Macular Degeneration in CNV Mouse Model. *International journal of molecular sciences* **17**, 10.3390/ijms17060895 (2016). 10.3390/ijms17060895 PMC4926429 27338342

[24] Turchinovich A, Zoidl G, Dermietzel R (2010). Non-viral siRNA delivery into the mouse retina *in vivo*. BMC ophthalmology.

[25] Ehrenberg, M. & Benny, O. Evolving multidimensional pharmacological approaches to CNV therapy in AMD. *Current eye research*, 1–8, 10.1080/02713683.2017.1385088 (2017). 10.1080/02713683.2017.1385088 29111834

[26] Campa C, Harding SP (2011). Anti-VEGF compounds in the treatment of neovascular age related macular degeneration. Current drug targets.

[27] Grunewald FS, Prota AE, Giese A, Ballmer-Hofer K (2010). Structure-function analysis of VEGF receptor activation and the role of coreceptors in angiogenic signaling. Biochim Biophys Acta.

[28] Kerbel RS (2008). Tumor angiogenesis. The New England journal of medicine.

[29] Nagy JA, Dvorak AM, Dvorak HF (2007). VEGF-A and the induction of pathological angiogenesis. Annu Rev Pathol.

[30] Kim KL, Suh W (2017). Apatinib, an Inhibitor of Vascular Endothelial Growth Factor Receptor 2, Suppresses Pathologic Ocular Neovascularization in Mice. Investigative ophthalmology & visual science.

[31] Schlaeppi JM, Wood JM (1999). Targeting vascular endothelial growth factor (VEGF) for anti-tumor therapy, by anti-VEGF neutralizing monoclonal antibodies or by VEGF receptor tyrosine-kinase inhibitors. Cancer metastasis reviews.

[32] Wang, W. & Lo, A. C. Y. Diabetic Retinopathy: Pathophysiology and Treatments. *International journal of molecular sciences* **19**, 10.3390/ijms19061816 (2018). 10.3390/ijms19061816 PMC6032159 29925789

[33] Bae JH (2017). Intravitreal itraconazole inhibits laser-induced choroidal neovascularization in rats. PloS one.

[34] Lou DA, Hu FN (1987). Specific antigen and organelle expression of a long-term rhesus endothelial cell line. In vitro cellular & developmental biology: journal of the Tissue Culture Association.

[35] Schneider CA, Rasband WS, Eliceiri KW (2012). NIH Image to ImageJ: 25 years of image analysis. Nature methods.

[36] Lambert V (2013). Laser-induced choroidal neovascularization model to study age-related macular degeneration in mice. Nature protocols.

[37] Sato M (2011). Identification of transcription factor E3 (TFE3) as a receptor-independent activator of Galpha16: gene regulation by nuclear Galpha subunit and its activator. The Journal of biological chemistry.

