# Supplementary material for: Activator of G-protein signaling 8 is involved in VEGF-induced choroidal neovascularization
Source: Sci Rep. 2019 Feb 7;9:1560. doi: 10.1038/s41598-018-38067-4 (PMC6367328; doi:10.1038/s41598-018-38067-4)
Supplement: Supplementary file 1 — Supplementary information [file 41598_2018_38067_MOESM1_ESM.pdf]

Activator of G-protein signaling 8 is involved in VEGF-induced choroidal neovascularization

[1]Hisaki Hayashi*, [1]Abdullah Al Mamun, [2]Masayuki Takeyama, [1]Aya Yamamura, [2]Masahiro Zako, [3]Rina Yagasaki, [3]Tsutomu Nakahara, [2]Motohiro Kamei, [1]Motohiko Sato*

[1]Department of Physiology, [2]Ophthalmology, Aichi Medical University, Nagakute, Japan
[3]Department of Molecular Pharmacology, Kitasato University School of Pharmacy, Tokyo, Japan

Corresponding authors:
Hisaki Hayashi, E-mail: h-hayashi@aichi-med-u.ac.jp
Motohiko Sato, E-mail: motosato@aichi-med-u.ac.jp

Supplementary Figures

Full length blot of Fig.1C

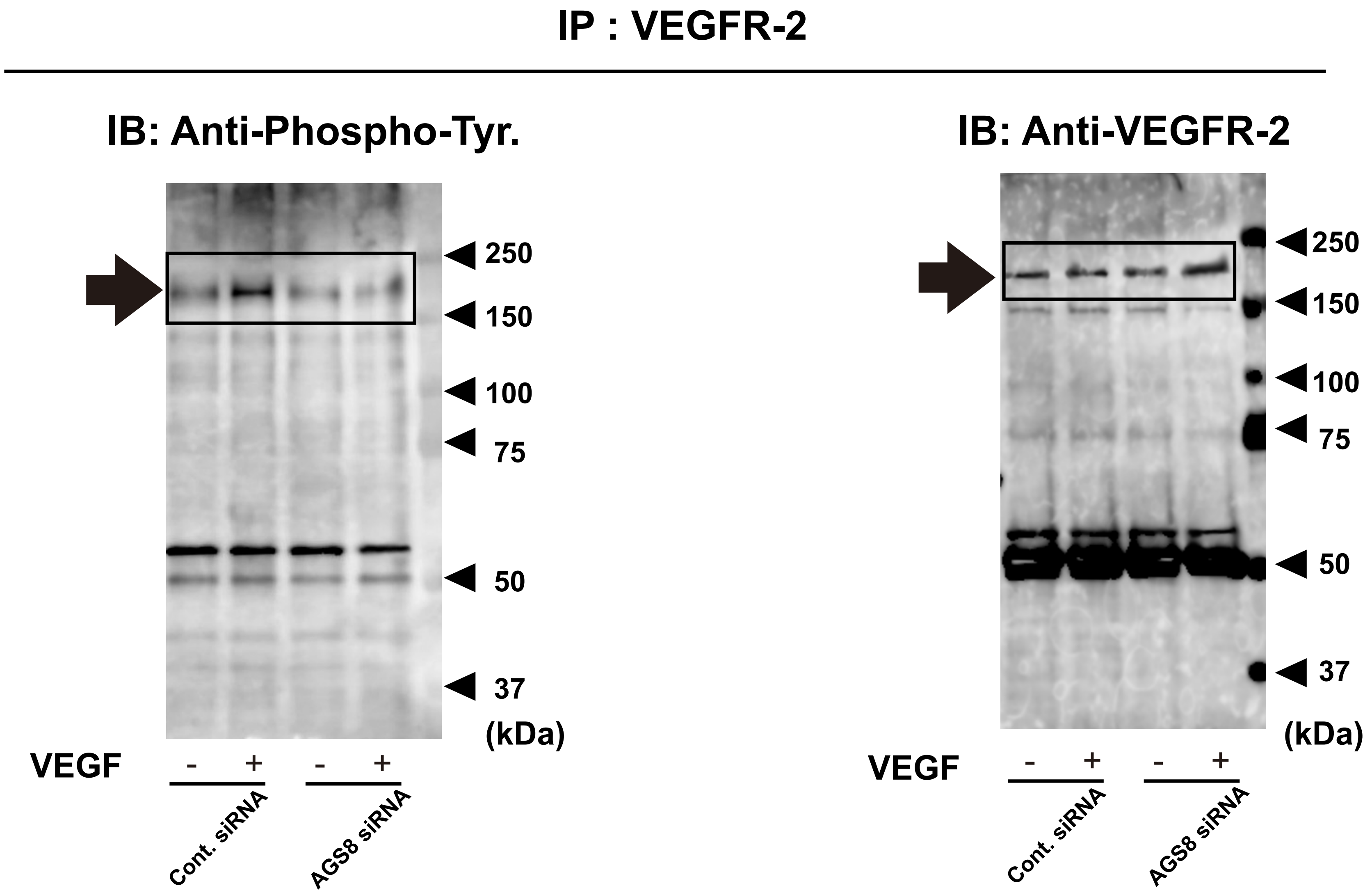

Supplementary Figure S1. Representative full-length immunoblots from Fig. 1C, showing tyrosine phosphorylation of VEGFR-2 (left panel) and expression of VEGFR-2 (right panel) in AGS8 knockdown in VEGF stimulated RF/6A cells. The black-line box in each blot referred to the cropped parts that are showed in the main article.

Full length blot of Fig.1D

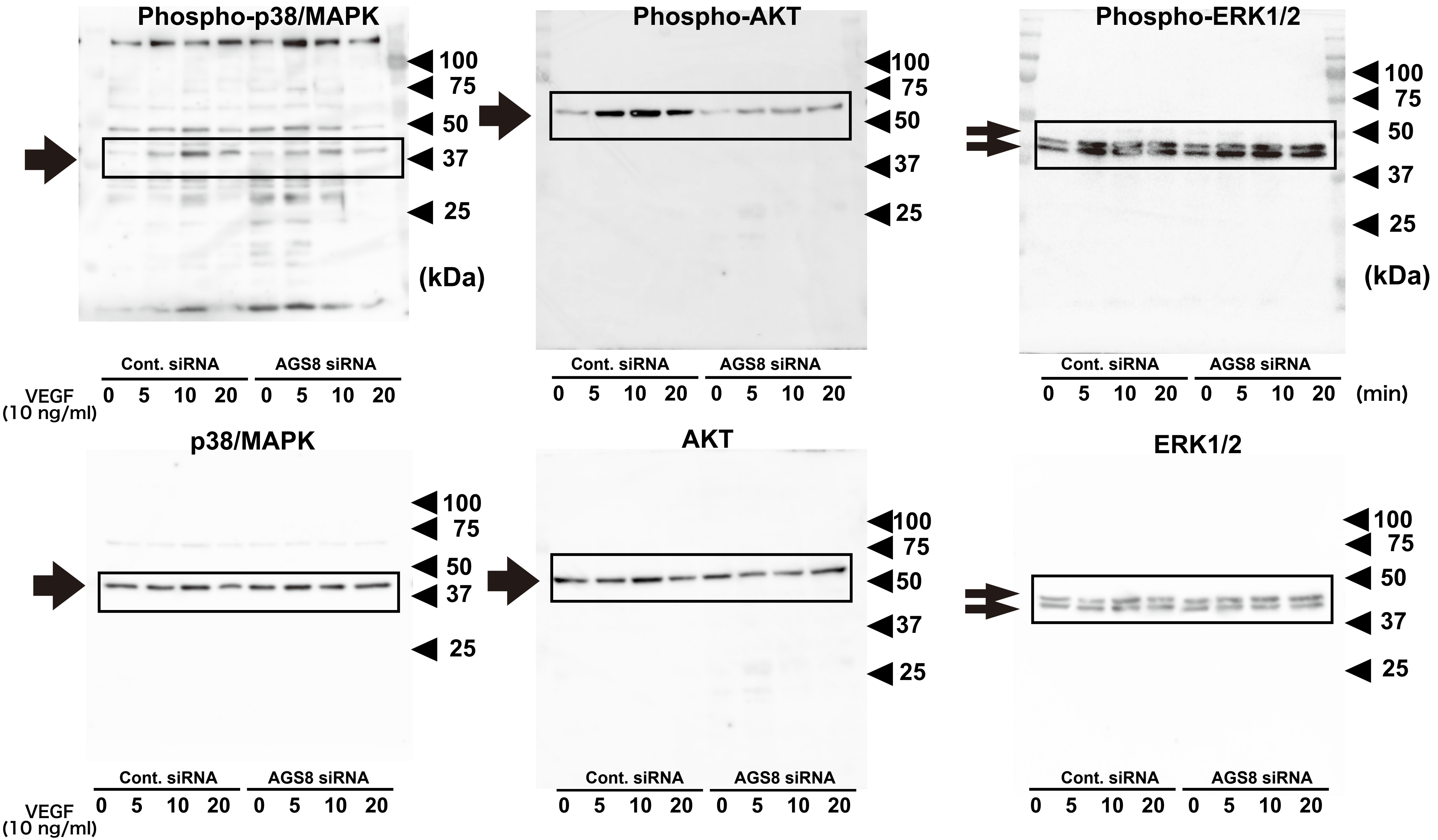

Supplementary Figure S2. Representative full-length immunoblots from Fig. 1D, showing tyrosine phosphorylation and expression of p38/MAPK, AKT, and ERK1/2 in AGS8 knockdown in VEGF stimulated RF/6A cells in time-dependent manner. The black-line box in each blot referred to the cropped parts that are showed in the main article.

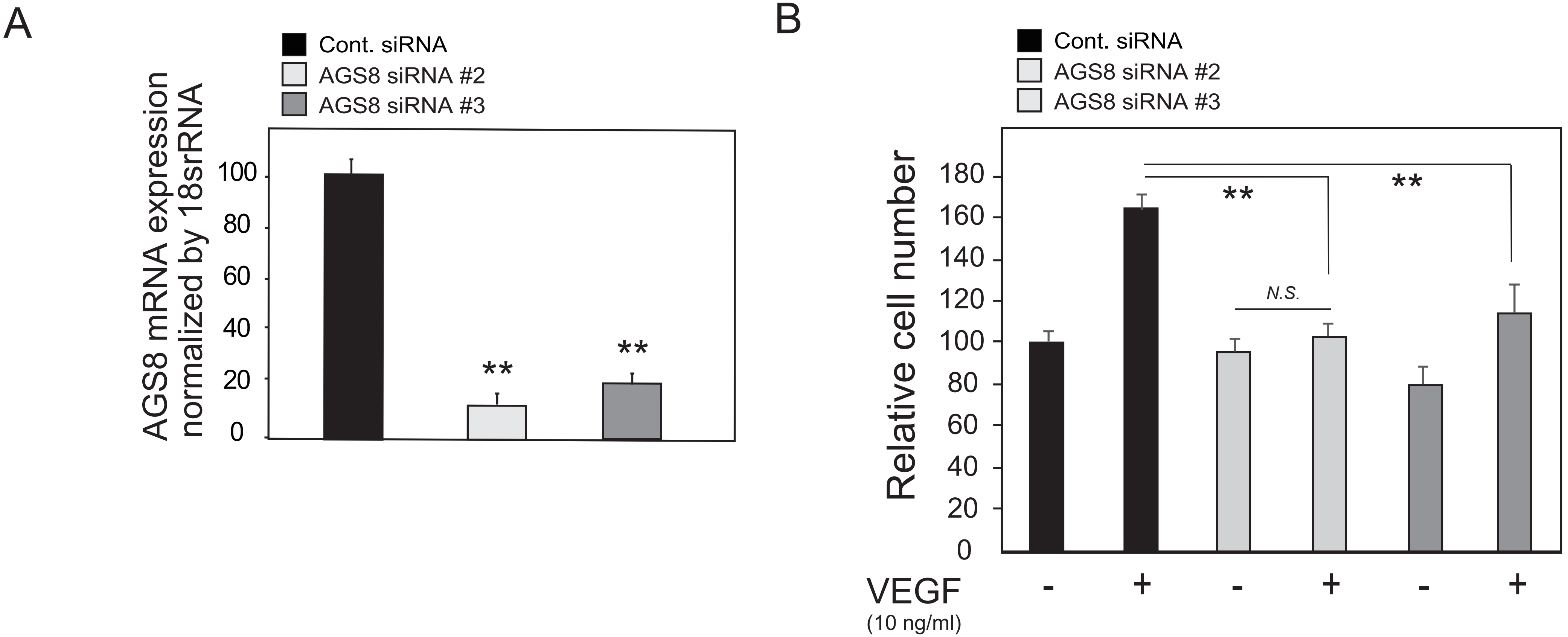

Supplementary figure S3. (A) Evaluation of AGS8 knockdown by real-time PCR. RF/6A cells were transfected with control siRNA, AGS8 siRNA#2, or AGS8 siRNA#3. After 48 h, AGS8 mRNA expression was analyzed by a real-time PCR assay. Data are expressed as the mean ± s.e.m. from 6 samples from 2 independent experiments. **$P < 0.01$ (unpaired t-test).

(B) Effect of AGS8 knockdown in VEGF-stimulated cells. After AGS8 knockdown in RF/6A cells by AGS8 siRNA#2 or AGS8 siRNA#3, the cells were stimulated with 10 ng/mL VEGF for 48 h and cell proliferation was analyzed with an MTT assay. Data are the mean ± s.e.m. from 5 independent experiments in quadruplicate. **$P < 0.01$ (two-way ANOVA with Tukey' s correction). N.S., not significant (unpaired t-test).

C

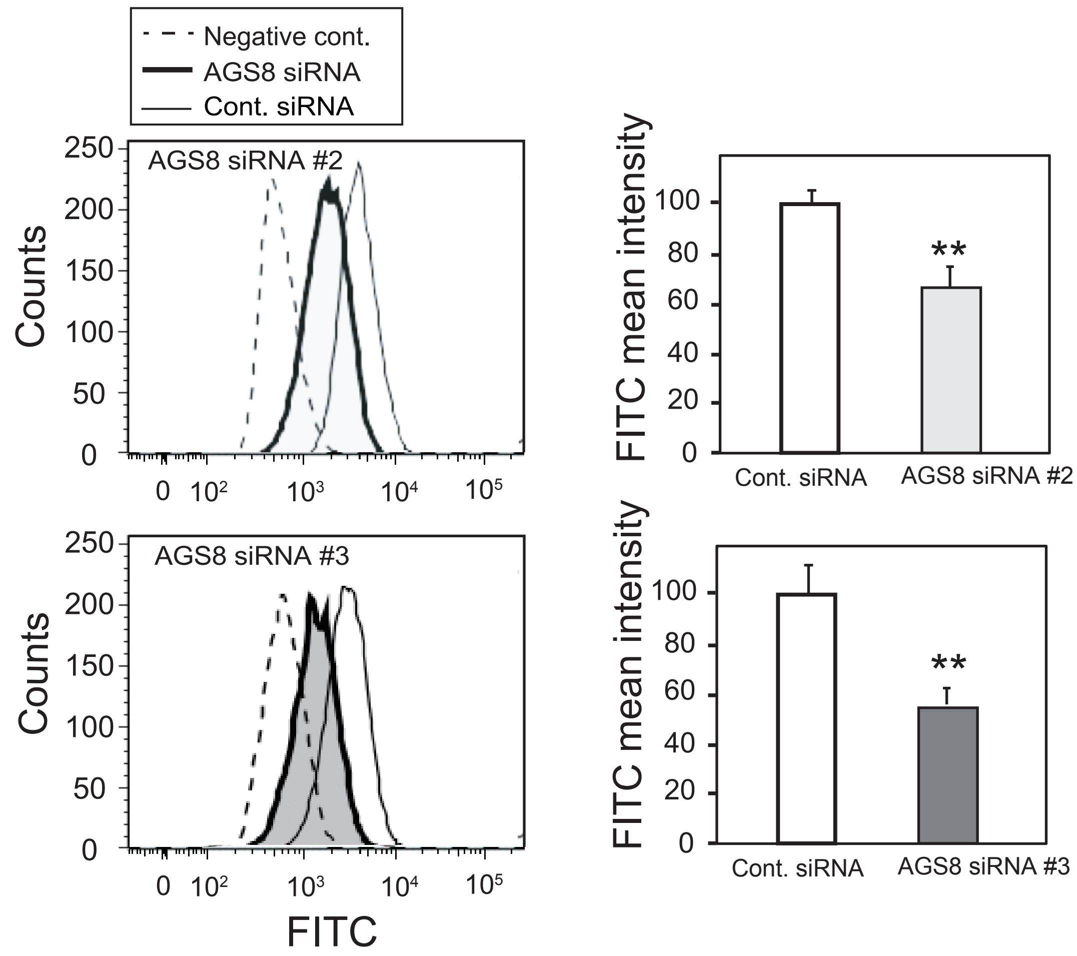

Supplementary figure S3. (C) VEGFR-2 expression on the cell surface is reduced by AGS8 knockdown. RF/6A cells were transfected with control siRNA, AGS8 siRNA#2 or AGS8 siRNA#3 and labeled with an fluorescein-conjugated anti-VEGFR-2 antibody, and flow cytometric analyses were performed as described in the Methods. The histograms represent cell counts (Y-axis, linear scale) versus fluorescein intensity (X-axis, log scale). Negative control indicates negative control cells treated with respective isotype human IgG labeled with fluorescein. Fluorescence mean intensity obtained from the histograms was quantified and shown in bar graphs (right panel). Data are the mean ± s.e.m from 4 experiments. **P < 0.01 versus control siRNA-treated cells (unpaired t-test).
